# The Limited Coverage of Facial Feminization Surgery in the United States: A Literature Review of Policy Constraints and Implications

**DOI:** 10.3390/jcm12165308

**Published:** 2023-08-15

**Authors:** Alejandra Aristizábal, Joseph M. Escandón, Pedro Ciudad, Oscar J. Manrique

**Affiliations:** 1 Division of Plastic and Reconstructive Surgery, Strong Memorial Hospital, University of Rochester Medical Center, New York, NY 14642, USA; 2 Department of Plastic, Reconstructive and Burn Surgery, Arzobispo Loayza National Hospital, Lima 15082, Peru

**Keywords:** facial feminization surgery approval, insurance coverage facial feminization surgery

## Abstract

There is a literature gap regarding facial feminization surgery (FFS) access and coverage. Our goal is to compile information from previous studies and assess the current policy landscape for these surgeries in the US. We also explored why some policies do not cover them, identify states with better coverage, and determine the most covered procedures. PubMed, Medline, Embase, and Scopus were searched for studies that reviewed policies on FFS coverage. Studies on surgical techniques or other gender-affirming surgeries (GASs) that did not involve FFS were excluded. Seven studies were included for analysis. In 2014, the Department of Human Health Services (HHS) lifted the transgender exclusion policy, leading to an increase in policies regarding GASs for both private and state insurance. However, there are differences in medical necessity requirements among policies, which may not align with the World Professional Association for Transgender Health (WPATH) criteria. States that prohibit exclusion tend to offer better coverage for FFS. These states are mainly located in the western and northeast regions, whereas states in the southern and middle east regions have less coverage. Among the procedures, chondrolaryngoplasty is the most covered, while facial and cervical rhytidectomy are the least covered. To enhance transgender care, it is crucial to reach a consensus on how to offer coverage for facial feminization surgery. However, there is a lack of adequate research on this topic, and there is a need for resources and tools to assess the results of FFS procedures. One significant constraint of this study is that it does not provide a systematic review of the literature.

## 1. Introduction

Gender-affirming surgeries (GASs) have increased by 115% from 2016 to 2017 as reported by The American Society of Plastic Surgeons (ASPS) [1]. This surge can be attributed to the progress of society towards inclusivity and national efforts to improve transgender healthcare [1]. In 2020, there were 16,353 GASs, representing a 12% increase from the previous year [2].

Facial feminization surgery (FFS) is a complex mixture of procedures performed on transfeminine people in which certain features are intervened to resemble feminine facial features; it has variations depending on the aim of the patient [3]. Some people view it as a cosmetic procedure, claiming that its purpose is to enhance appearance rather than being medically necessary to mitigate gender dysphoria symptoms [4].

This paper aims to examine the current state of facial feminization surgery coverage in the United States, its existing constraints, and the factors that contribute to its limited availability.

For a long time, the transgender community has faced barriers to accessing medical care. Despite efforts to improve the situation, patients in several states still have to contend with denials of procedures due to medical necessity criteria and a shortage of healthcare providers [4].

It is important to discuss this topic to address the healthcare system debt with the Lesbian, Gay, Transgender, Bisexual, Queer/Questioning, Intersexual, Asexual, and more (LGTBQIA+) community. However, it is important to avoid the misconception that every patient undergoing gender transition desires to have surgery [1].

Within the last decade, there have been efforts to improve access to healthcare, such as the “affordable care act”. This has had a significant impact on plastic surgery, including pediatric craniofacial surgery, breast reconstruction, and gender affirmation surgery [5].

As we researched this topic, we discovered various studies on the extent of FFS coverage and the obstacles that exist within the healthcare system to accessing these procedures. Our aim is to gather and analyze this information to offer a clearer picture of the current scenery.

## 2. Materials and Methods

PubMed, Medline, Scopus, and Embase were searched using the terms “facial feminization surgery coverage,” “facial feminization insurance,” and “facial feminization cost” without a year filter. The search was performed from April to June 2023.

We found a total of 39 articles with this search; after eliminating duplicates, 20 articles were left.

Studies reviewing coverage for facial feminization surgery, either commercial insurance policies or state policies, payer status, and procedures covered were included. Seven articles fulfilled the inclusion criteria, see Table 1.

**Figure 1:**
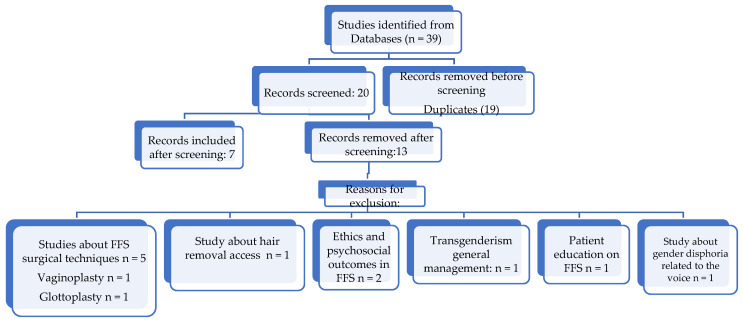
Facial Feminization Surgery Studies Database Search.

Papers reviewing surgical techniques for facial feminization surgery, body feminization surgery, vaginoplasty, and voice feminization surgery without reviewing costs, coverage, or insurance policies for facial feminization surgery were excluded. One paper reviewing coverage for hair removal was excluded as it solely focused on a non-surgical procedure and did not cover any other facial surgeries. We excluded a paper on voice feminization surgery from our review of facial feminization surgeries because, although it is a gender-affirming surgery, it does not involve the face. We included papers that discuss policies related to facial feminization surgeries. Some of these papers also touch upon topics such as hair removal and voice surgery, but they were included because they cover facial feminization surgeries as well. Thirteen articles were excluded from the analysis. The assessment of each record was performed manually by one reviewer, no automation tools were used for this (Figure 1).

## 3. Results

Facial feminization surgery has been around for almost 40 years [3,13]. Ousterhout defined it as improving facial aesthetics through bony contouring of the craniofacial skeleton, although he studied it at first in females with masculine characteristics [13]. In 1981, The US Department of Human Health Services (HHS) excluded transgender surgical treatments from insurance coverage [8]. In 2010, the “Affordable Care Act” bill was signed and in 2014, the HHS’s previous exclusion was overturned [5,8]. Since then, patients seeking gender-affirming surgery (GAS) have dramatically increased [8,14]. Over the past 20 years, Fortune 500 companies such as Starbucks and other major technology companies have increased their coverage for gender-affirming care benefits by 1700% [6,7,15]. Contrasting with the US transgender care approach, Sweden acknowledged in 2015 that FFS should be included in gender-affirming care for individuals with gender dysphoria [4].

### 3.1. Commercial Policies

Insurance companies started offering policies for gender-affirming care after the HHS transgender surgical exclusion was lifted [6]. However, 10% of companies do not offer coverage or have a policy for GAS [6]. 

Ngaage et al. identified commercial insurance companies with policies related to gender-affirming surgery [6]. They included 92 companies representing 90% of the market share. From these, 86 companies had GAS policies, 2 of them had no coverage policies, and 6 did not have any policies [6] (Table 1). Despite the creation of more GAS policies compared to the period before the 2014 HHS prohibition overturn, they found that most of the companies increased by almost 40% the medical necessity criteria non-related to WPATH standards to access their GAS policies [6]. Even so, there was a shift in coverage for the so-called ancillary procedures such as FFS (policy inclusion vs. exclusion 4–16% *p* = 0.0088), hair transplantation (policy inclusion vs. exclusion (0–12% *p* = 0.0003), and chondrolaryngoplasty (policy inclusion vs. exclusion 2–15% *p* = 0.0051) Table 2.

In another study, Ngage et al. found that of the 61 commercial insurers with GAS policies, 14 (23%) companies had favorable policies for at least one facial procedure in the 2018–2019 period [7] (Table 2).

Gadkaree et al. investigated the FFS policies of 150 companies engaged with large-group commercial insurers, representing the highest enrollment per state in 2019. One company did not have any public policy and from the remaining 149, only 27 (18%) had favorable policies (covered at least 1 FFS procedure), and a mean of 5.15 procedures were covered by policy [8].

Gorbea et al. reviewed the top 57 premium companies that accounted for 81% of all insurance premiums; excluding nonprimary insurance companies, 49 remained for analysis [9]. Out of the 45 companies surveyed, 92% had published policies regarding transgender coverage. Among these, 51% described FFS as cosmetic, while 36% considered it medically necessary. Six companies did not classify it as either. A total of 60% of the companies excluded all FFS procedures, while 40% offered some level of FFS coverage [9].

Almazan et al. reviewed the insurance policies for GAS from the three largest insurers by market share in each state, including 122 GAS policies from which 24.7% covered FFS procedures [10].

### 3.2. Coverage by State

The US government of San Francisco became the first employer offering transgender healthcare back in 2001 [10]. In 2013, California enacted a prohibition against transgender insurance exclusions and since then many states have followed [10]. This prohibition prevents insurance companies from creating blanket exclusions for services related to transgender transitional care [7,8,10].

California has been a pioneer in transgender rights; this allowed that 90% of patients were approved, under insurance, for FFS at UCLA during the 2018–2020 period [11]. Nonetheless, the road to authorization varies among patients according to their type of insurance. Patients under public insurance by Medical (n = 13), Medicare (n = 4), and private California-insured plans (n = 9) had an authorization time of 1.1 months, whereas patients under private insurance (non-California insured) or job-based self-insurance plans (employee retirement income security act “ERISA”) plans had an authorization time of 6 months and were more likely to undergo multiple appeals and denials; this is because they are not covered by California anti-transgender exclusion legislation [11] (Table 3).

Most of the states prohibiting transgender insurance exclusions are in the west and northeast, and the top insurers in these states tend to have policies that offer more GAS services; in fact, most of the FFS procedures occurred in these two regions [10,12]. However, these exclusion prohibitions do not mandate specific procedure coverage [10]. 

A review by Gorbea et al. Examined Medicaid policies in the US and discovered that 30 states have specific policies for Medicaid coverage for transgender or nonbinary patients. Out of these, 18 states provide coverage for GAS, while 13 states do not cover any [9]. Out of the 18 mentions of GAS coverage, only 7 referred to FFS. However, only 3 of those provided coverage for any FFS procedure, while the remaining 4 considered it to be a cosmetic procedure rather than a medical necessity [9], see Table 3.

Connecticut, Massachusetts, and Washington provide extensive FFS coverage (multiple procedures) for people enrolled in Medicaid since it is considered potentially medically necessary [9]. In other states such as Maine, New Jersey, and Michigan, Medicaid contractors must provide GAS coverage; however, there are no specifics on which services must be included [9]. Maryland considers thyroid chondroplasty medically necessary [9].

The coverage of ancillary procedures for the face, neck, and hair may vary, but the coverage for genital-affirming surgeries is similar in most states [10]. Phalloplasty and vaginoplasty were covered in 95.7% of the insurance policies reviewed from states with transgender exclusion prohibitions, while it was covered by 93.6% and 96.2%, respectively, in states without these prohibitions [10] (Table 3). Interestingly, in Oklahoma, despite not having exclusion prohibitions for private insurers, 94% of the insured population has access to these procedures; according to Ngage et al., this might be because the largest insurer there, Health Care Service Corporation (HCSC), has a large market share in other states that do have a prohibition for GAS exclusions [7].

Another issue is that even if the state has somehow good access to GAS, 22 states do not have professionals specialized in GAS, even fewer in FFS, and many are only trained to modify soft tissue structures [4].

### 3.3. Procedures Covered

Previous studies analyzed policies related to gender affirmation surgery, including FFS procedures, and identified which ones are covered [7,8,9,10,11] (Table 4).

Multiple studies found that chondrolaryngoplasty is the most frequently covered procedure and is deemed medically necessary in certain policies. It is consistently covered in 30 states [7,8,9,10,11], followed by procedures such as forehead contouring, blepharoplasty, rhinoplasty, and mandible-related procedures, whereas procedures typically considered related to rejuvenation are less covered, such as facial rhytidectomy or cervicoplasty [7] (Table 4).

Since insurance policies commonly deny FFS procedures, many patients are self-payers. According to Hauc et al., data from the National Inpatient Sample (NIS) were analyzed between 2008 and 2017 to determine the number of patients who underwent gender affirmation surgery [12]. Out of the 1215 patients who had other GASs, 110 also had FFS. The breakdown by payer was as follows: 35 patients (31.8%) had Medicaid, 30 patients (27.3%) had private insurance, and 40 patients (36.4%) paid for the procedure themselves (Table 4).

Despite FFS rising across the country, cost is still a barrier for transgender patients [12]. In a cross-sectional study by Firouzi et al., it was found that out of the 887 individuals surveyed, 383 identified as transwomen. Only 7% of these individuals had undergone FFS, while 74% expressed an interest in the procedure and 67% expressed a desire to undergo it [12]. According to these data, 43% of them reported that the cost was the main obstacle preventing them from accessing FFS, while 28% said that cost was a barrier to accessing bottom surgery [12] (Table 4).

The California experience showed how the authorization process was more costly for patients getting delayed by their insurance [11]. Patients with a delayed insurance appeal process had 22-fold higher cost and patients that ended up in denial had a cost 26 times higher than that of patients who had standard authorization [11].

### 3.4. Medical Necessity Criteria

Facial feminization surgery is one of the most commonly denied GAS procedures based on the belief that it is merely cosmetic and not a medical necessity [11], while on the other hand, commercial insurers consider medical necessity procedures, genital reconstruction in 100%, gender-affirming mastectomy in 98%, and breast augmentation in 62% of their policies [9].

Despite nationwide efforts to increase access to GAS, certain insurance companies still have medical necessity criteria that create barriers to access. The World Professional Association for Transgender Health has proposed medical criteria for GAS in their Standards for Transgender Care 8th version [16] (Table 5). They have addressed the cosmetic vs. medical necessity debate about FFS, stating that it is not a cosmetic procedure [4,9]. According to Gorbea et al., 74% of policies cite the WPATH standards of care; nevertheless, this did not correlate with FFS coverage by them [9]. However, many companies have created their own interpretation of these criteria or have come up with their own policies [9] (Table 6).

As an example, Gadkaree et al. found that from the 149 policies reviewed, 26 had published medical necessity criteria; however, many had criteria outside the WPATH standards such as regular visits with a mental health provider (19/26), a referral from a mental health provider (18/26), age majority (21/26), and 12 months of congruent experience (19/26) [8]. A name change was not required in any of the policies [8] (Table 6).

From the 61 insurance companies with ancillary procedures established policies, one in five (n = 12) stated medical necessity criteria for ancillary procedures [7] (Table 6).

## 4. Discussion

Gender dysphoria causes distress by the incongruence between one’s gender and body; however, it can be effectively treated with gender affirmation surgery (GAS) [17]. Transgender individuals often opt for these procedures to alleviate gender dysphoria, which can lead to depression, anxiety, and self-destructive behavior if left untreated. Several reports have shown that the transgender population has a suicide rate of 40% [4]. Facial feminization surgery enables patients to present themselves as the gender they identify with, leading to improved quality of life, and reduced mental distress, anxiety, discrimination, and violence toward them.

In the US, the overturn of the insurance coverage prohibition for gender-affirming surgery is still very new; in fact, all the studies reviewed were conducted after 2017 [5,8]. Despite the increased creation of policies for GAS coverage by 56% after the overturn, insurance companies also increased medical necessity criteria by 40% to access their policies, many opposed the current World Professional Association for Transgender Health WPATH standards criteria [6,9].

It is considered a positive outcome that the majority of policies now recognize Gender-affirming surgery as a crucial medical requirement for individuals who identify as transgender [10]. Unfortunately, at the same time, the so-called ancillary procedures of the face, neck, and hair transplant-related suffered a significant increase in their exclusion rate [6]. This appears to be contradictory that chondrolaryngoplasty, a procedure for facial feminization, is widely covered across the country, and in some states, it is even considered a medical necessity [7,8,9].

Judging by the policy coverage limitations, FFS is considered cosmetic by many people despite being recognized as medically necessary by WPATH standards of care 8th edition [16].

One reason for this belief may be due to a focus on genital-related transitions, which some may perceive as the only or most crucial surgical step [4,10,18,19,20]. The face is arguably as important as the genitals because it plays a significant role in how people see you and it is the first impression that others will perceive from the patient during daily social interactions. During most human interactions, genitals and other parts of the body are usually hidden, such as the breasts; however, breast augmentation procedures have a broader policy coverage than facial feminization surgery [4,10,21].

Facial procedures are often geared towards achieving beauty standards from a cisgender perspective, which is reflected in insurance policies, not the principles behind transgender patients. However, individuals with gender dysphoria experience ongoing distress due to the mismatch between their gender identity and the sex assigned at birth. FFS can alleviate this distress and greatly improve the lives of these patients [10].

For many transfeminine people, FFS is a desired procedure to aid with their transition. However, the high cost remains a major obstacle [1]. The benefits of GAS have been discussed in the literature regarding the improvement of patients’ lives and reduction of healthcare costs [17,18,19,20]. Treating gender dysphoria in transgender patients through GAS has been shown to decrease depression, violence, and even suicide [17,18,19]. This makes it a cost-effective procedure that should be covered [6]. The California experience demonstrated that when patient authorization processes are delayed, it leads to increased expenses for the healthcare system [11,22].

It is worth noting that FFS (facial feminization surgery) coverage varies across the country. States with transgender exclusion prohibitions tend to offer better coverage for FFS than those without. The west and northeast regions are generally more transgender care friendly, both in terms of policies and the availability of trained surgeons, which may explain the higher coverage [6,12]. It is currently unknown how the increasing number of state laws that limit transition care services for minors will affect policy soon. Florida, Arizona, and Nebraska have already banned GAS for minors as an example of this trend and many others have banned hormone therapy as well [23].

There are some points that would improve FFS coverage in the near future. It is important to medically define which procedures are included in FFS since WPATH does not define it clearly, leaving a gray zone for insurance companies to interpret it.

Conscientize people about the approach of GAS and how its final goal is not to achieve beauty but to improve gender dysphoria.

Incentivize the upcoming generation of plastic surgeons to learn about GAS, FFS, and other procedures for the transgender community. In addition, we should conduct more research to determine the benefits of offering these procedures. This will help patients reintegrate into society and reach their full potential, leading to greater productivity.

## 5. Conclusions

Despite many positive reports in the literature regarding gender-affirming care, it is still immature in the US. Facial feminization surgery is a costly and intricate procedure. Although it has become more accessible in the past decade, it is still unaffordable for many transgender patients.

Our study highlights the necessity for improved policies to prevent discrimination in healthcare. It is important to reach a consensus on the coverage of procedures and revisional surgery and establish centers of excellence for transgender care. However, the major weakness of our paper is not having a real cost-effect analysis of each procedure, and its direct implications on each one of the patients treated. For that reason, some people might see this as a purely cosmetic procedure.

Currently, the lack of tools available to accurately measure the objective outcomes and benefits of facial feminization surgery are still needed to promote and expand current healthcare policies that can give equal access to the transgender population.

## Figures and Tables

**Table 1:** Studies included.

Year	Author	Study	Location	Study Type	Observation Period	Summary	Outcomes			
							Policy Coverage	State Policy	Procedures Covered	Medical Necessity Criteria
2021	Ngaage et al. [6]	Gender-Affirming Health Insurance Reform in the United States	United States	Cross sectional	2007–2018	To examine changes in insurance policies after the Department of Health and Human Services prohibited insurance discrimination of transgender individuals in 2014.	x			
2020	Ngage et al. [7]	Gender Surgery Beyond Chest and Genitals: Current Insurance Landscape	United States	Cross sectional	12/2018–02/2019	To assess the frequency of coverage provision for ancillary transition-related surgeries through a cross-sectional analysis of US insurance policies.	x		x	x
2021	Gadkaree et al. [8]	National Variation of Insurance coverage For Gender affirming Facial Feminization Surgery	United States	Cross sectional	2018–2019	To determine insurance coverage and ease of finding policy information for FFSs, and analyze differences based on state advocacy.	x		x	x
2021	Gorbea et al. [9]	Insurance Coverage of Facial Gender Affirmation Surgery: A Review of medicaid and commercial insurance	United States	Systematic review	01/2020–05/2020	To review state policies on transgender care for 50 states.	x	x	x	x
2020	Almazan et al. [10]	Associations Between Transgender Exclusion Prohibitions and Insurance Coverage of Gender-Affirming Surgery	United States	Cross sectional	05/2019–08/2019	To evaluate coverage of gender-affirming surgery between states that do and do not have prohibitions against explicit transgender exclusions in private insurance.	x	x	x	x
2021	Hu et al. [11]	Facial Feminization Surgery Under insurance, The university of California, LA, Experience	LA, California	Case series	2018–2020	To assess time and costs of the FFS insurance coverage and authorization process in California.		x	x	
2022	Hauc et al. [12]	Limited Access to Facial Feminization Geographically Despite Nationwide Expansion of Other Gender-Affirming Surgeries	United States	Case series	2008–2017 for all GAS FFS data only through 2015–2017	To estimate trends in GAS and access to FFS depending on geographical zone and primary payer in the US.		x		

x: outcome reviewed in each paper.

**Table 2:** Commercial policies.

Year	Author	Study	Study Type	Observation Period	Methods	Companies Included	Policies	Any Policies for FFS
2021	Ngaage et al. [6]	Gender-Affirming Health Insurance Reform in the United States	Cross sectional	2007–2018	Insurance providers were selected based on company market share. We conducted a Web-based search and telephone interviews to identify policies related to GAS.	92	Stated GAS: 86 No coverage: 2 No GAS policy: 6	Not assessed
2020	Ngage et al. [7]	Gender Surgery Beyond Chest and Genitals: Current Insurance Landscape	Cross sectional	12/2018–02/2019	The top 3 insurers from each state were cross-referenced with market share. Thus, 63 insurance companies were included, representing 80% of the market share.	63	Stated GAS: 61 No GAS policy: 2	14 (23%)
2021	Gadkaree et al. [8]	National Variation of Insurance coverage For Gender affirming Facial Feminization Surgery	Cross sectional	2018–2019	The top three largest commercial health plans per state based on 2019 enrollment data were included.	150	Stated GAS: 149 no public policy: 1	27 (18%)
2021	Gorbea et al. [9]	Insurance Coverage of Facial Gender Affirmation Surgery: A Review of Medicaid and commercial insurance	Systematic review	01/2020–05/2020	State policies on transgender care were collected for 50 states. The largest companies were identified using the national association of insurance commissioners’ market.	50	Stated 45 No GAS policy 4 No public policy 1	27 (60%) excluded all FFS procedures, 18(40%) offered some degree of coverage
2020	Almazan et al. [10]	Associations Between Transgender Exclusion Prohibitions and Insurance Coverage of Gender-Affirming Surgery	Cross sectional	05/2019–08/2019	Insurance policies for gender-affirming surgery were obtained from the three largest insurers, by market share, in each state.	124	Stated GAS: 95 No available policy: 29	24.7%

**Table 3:** Policies according to state.

Year	Author	Study	Type	Observation Period	Methods	Coverage Findings	FFS Related Policies
2021	Hu et al. [11]	Facial Feminization Surgery Under insurance, The university of California, LA, Experience	Case series	2018–2020	FFS consults (n = 40) at UCLA were reviewed for time and cost to authorization.	1. Medical (n = 13), Medicare (4), and private California insured plans (9) had an authorization time of 1.1 months. 2. Private insurance (n = 10) 7 months for authorization 3. Private insurance from ERISA (n = 4) denied.	90% of 40 patients were accepted for one stage FFS in UCLA between 2018 and 2020.
2022	Hauc et al. [12]	Limited Access to Facial Feminization Geographically Despite Nationwide Expansion of Other Gender-Affirming Surgeries	Case series	2008–2017 for all GAS FFS data only through 2015–2017	Subtracted data from NIS National inpatient sample and reviewed the primary payer for GAS and FFS based on ICD dx on hospital stays.	Most FFS procedures occurred. 1. West (50%) 2. Northeast 35 (31.8%) 3. South 15 (13.6%) 4. Midwest (5, 4.8%)	1. Connecticut, Massachusetts, and Washington cover FFS extensively without case-by-case clause. 2. Maryland, California, and Oregon cover certain procedures. 3. Colorado started to cover jaw, cheek, and facial bone remodel in 2023.
2020	Ngage et al. [7]	Gender Surgery Beyond Chest and Genitals: Current Insurance Landscape	Cross sectional	12/2018–02/2019	The top 3 insurers from each state were cross-referenced with market share. Thus, 63 insurance companies were included, representing 80% of the market share.	States with greater coverage are the ones with transgender exclusion prohibitions in private insurance plans.	States without any ancillary procedure coverage = 19 States with favorable coverage for FFS = 26
2021	Gorbea et al. [9]	Insurance Coverage of Facial Gender Affirmation Surgery: A Review of Medicaid and commercial insurance	Systematic review	01/2020–05/2020	State policies on transgender care were collected for 50 states. The largest companies were identified using the national association of insurance commissioners’ market.	States with GAS policies: 30 GAS coverage: 18 Denied Coverage: 13	FFS policies: 7 Coverage: 3 Denied coverage 4 Extensive coverage; Washington, Connecticut, Massachusetts
2020	Almazan et al. [10]	Associations Between Transgender Exclusion Prohibitions and Insurance Coverage of Gender-Affirming Surgery	Cross sectional	05/2019–08/2019	Insurance policies for GAS obtained from the three largest insurers, by market share, in each state.	Policies based in states with TG insurance prohibitions: 46 (37.1%) Policies in states that do not prohibit TG exclusions in private insurance: 78 (62.9%)	1. FFS covered by 19.6% of policies in states with TG exclusion prohibitions. 2. 5.1% of policies in states without exclusion prohibitions

**Table 4:** Covered FFS procedures.

Year/Author	Study	Covered Procedures	Covered Procedures from Most Covered to Least	% of Policies That Cover the Procedure
Gorbea et al., 2021	Insurance Coverage of Facial Gender Affirmation Surgery: A Review of Medicaid and commercial insurance. [9]	1. Most covered FGAS by commercial insurers 2. Was described as a medically necessary aspect of transgender care in 100% of the commercial policies reviewed 3. Variable coverage and recognition as medical necessity	Thyroid chondroplasty Genital reconstruction Forehead contouring Mandibular contouring Rhinoplasty Blepharoplasty Facelift Genioplasty Browlift Voice modification. Cheek augmentation Cervical rhytidectomy	60% 100% 13% 13% 13% 13% 13% 11% 11% 9% 9% 9%
Gadkaree et al., 2021	National Variation of Insurance Coverage for Gender Affirming Facial Feminization Surgery. [8]	1. Chondrolaryngoplasty with preauthorization by 78% (n = 21) of favorable policies. -Nine companies provided coverage for chondrolaryngoplasty only	Chondrolaryngoplasty Rhinoplasty Forehead contouring Blepharoplasty Mandible shaping Facelift Hair transplant Liposuction Browlift Cheeks Neck lift Lip enhancement Otoplasty Chin reduction	78% 44% 44% 44% 44% 44% 40% 33% 33% 29% 18% 18% 11% 3%
Almazan et al., 2020	Associations Between Transgender Exclusion Prohibitions and Insurance Coverage of Gender-Affirming Surgery. [10]	Phalloplasty and vaginoplasty covered in 95% insurers in states that prohibit transgender exclusions--> coverage is very consistent through private insurers even in states that do not prohibit TG exclusions.	Phalloplasty Vaginoplasty Mastectomy Augmentation mammoplasty FFS FMS Thyroid chondroplasty (30% in states the prohibit TG exclusion)	Policies of top insurers in states that do not prohibit transgender exclusions. 95% 95% 95% 52% 5.1% 7.7% Policies of top insurers in states that prohibit transgender exclusions. 95% 95% 97% 65% 19.6% 30%
Ngaage et al., 2020	Gender Surgery Beyond Chest and Genitals: Current Insurance Landscape. [7]	1. 60% (n = 30) of states have favorable policies for chondrolaryngoplasty across the US 2. n = 14 have favorable coverage for facial procedures 23% (n = 14) of companies covered at least 1 FFS procedure. blepharoplasty 8% always covered and 13% on case-by case basis	Chondrolaryngoplasty Blepharoplasty Browlift Cervicoplasty Cheek implants Facial bone reconstruction Forehead contouring. Genioplasty Lip enhancement Mandible reconstruction Rhinoplasty Rhytidectomy	28% 8% 4.9% 1.6% 6.5% 4.9% 3.2% 3.2% 1.6% 4.9% 6.5% 6.5%
Hu et al., 2022	Facial Feminization Surgery Under insurance, The university of California, LA, Experience. [11]		Brow lift Forehead Fat graft Rhinoplasty with osteotomies Two-piece osseous genioplasty Reduction of mandibular angles Tracheal shave Upper lip lift Canthopexy Zygoma reduction	100% 95% 92.5% 90% 82.5% 72.5% 55% 55% 5% 2.5%

**Table 5:** Medical necessity criteria according to WPATH.

Criteria for Surgery According to WPATH (World Professional Association for Transgender Health) [16]
a. Gender incongruence is marked and sustained. b. Meets diagnostic criteria for gender incongruence prior to gender-affirming surgical intervention in regions where a diagnosis is necessary to access healthcare. c. Demonstrates capacity to consent for the specific gender-affirming surgical intervention. d. Understands the effect of gender-affirming surgical intervention on reproduction and they have explored reproductive options. e. Other possible causes of apparent gender incongruence have been identified and excluded. f. Mental health and physical conditions that could negatively impact the outcome of gender-affirming surgical intervention have been assessed, with risks and benefits having been discussed. g. Stable on their gender-affirming hormonal treatment regime (which may include at least 6 months of hormone treatment or a longer period if required to achieve the desired surgical result, unless hormone therapy is either not desired or is medically contraindicated)

**Table 6:** Medical necessity criteria.

Author	Study	Medical Necessity Criteria	
Gadkaree et al. 2020 [8]	National Variation of Insurance Coverage for Gender Affirming Facial Feminization Surgery	Available for 26 companies	
		1. Gender dysphoria diagnosis 2. Age of majority 3. 12 months of congruent experience 4. Regular visits with a mental health provider 5. Referral from a mental health provider	1. n = 25 2. n = 21 3. n = 19 4. n = 19 5. n = 18
Gorbea et al. 2021 [9]	Insurance Coverage of Facial Gender Affirmation Surgery: A Review of Medicaid and commercial insurance	Medical necessity criteria for FFS stated by each company	
Ngaage et al. 2020 [7]	Gender Surgery Beyond Chest and Genitals: Current Insurance Landscape	Available for 12 companies	Policies
		Age > 18 years	7
		Age is not a requirement	5
		Hormone therapy > 12 months	7
		Hormone therapy not a requirement	5
		Continuous living in congruent gender role for 12 m	6
		Continuous living in congruent gender role for 24 m	1
		Continuous living in congruent gender role not required	5
		One referral from health professional	3
		Two referrals from health professional	4
		Referral from health professional not required	5
Almazan et al. 2020 [10]	Associations Between Transgender Exclusion Prohibitions and Insurance Coverage of Gender-Affirming Surgery	Medical necessity criteria for FFS stated by the company	

## Data Availability

The results reported in this study can be found in the referenced articles.
